# Chronic Stress and Adolescents’ Mental Health: Modifying Effects of Basal Cortisol and Parental Psychiatric History. The TRAILS Study

**DOI:** 10.1007/s10802-014-9970-x

**Published:** 2015-01-25

**Authors:** Anna Roos E. Zandstra, Catharina A. Hartman, Esther Nederhof, Edwin R. van den Heuvel, Andrea Dietrich, Pieter J. Hoekstra, Johan Ormel

**Affiliations:** 1 Department of Psychiatry, University of Groningen, University Medical Center Groningen, Groningen, The Netherlands; 2 Department of Epidemiology, University of Groningen, University Medical Center Groningen, Groningen, The Netherlands; 3 Faculty of Mathematics and Natural Sciences, University of Groningen, Groningen, The Netherlands; 4 Department of Mathematics and Computer Science, Eindhoven University of Technology, Eindhoven, The Netherlands

**Keywords:** Chronic psychosocial stress, Long-term difficulties, Parental psychiatric history, Externalizing and internalizing problems, Basal cortisol upon waking, Adolescence

## Abstract

Large individual differences in adolescent mental health following chronic psychosocial stress suggest moderating factors. We examined two established moderators, basal cortisol and parental psychiatric history, simultaneously. We hypothesized that individuals with high basal cortisol, assumed to indicate high context sensitivity, would show relatively high problem levels following chronic stress, especially in the presence of parental psychiatric history. With Linear Mixed Models, we investigated the hypotheses in 1917 Dutch adolescents (53.2 % boys), assessed at ages 11, 13.5, and 16. Low basal cortisol combined with the absence of a parental psychiatric history increased the risk of externalizing but not internalizing problems following chronic stress. Conversely, low basal cortisol combined with a substantial parental psychiatric history increased the risk of internalizing but not externalizing problems following chronic stress. Thus, parental psychiatric history moderated stress- cortisol interactions in predicting psychopathology, but in a different direction than hypothesized. We conclude that the premise that basal cortisol indicates context sensitivity may be too crude. Context sensitivity may not be a general trait but may depend on the nature of the context (e.g., type or duration of stress exposure) and on the outcome of interest (e.g., internalizing vs. externalizing problems). Although consistent across informants, our findings need replication.

**Electronic supplementary material:**

The online version of this article (doi:10.1007/s10802-014-9970-x) contains supplementary material, which is available to authorized users.

Although chronic psychosocial stress is a well-known risk factor for both internalizing (mood and anxiety) and externalizing (behavioral) problems (Grant et al. 2004), interindividual differences in outcome are large. In turn, psychopathology may influence stress levels (Burke et al. 2002), providing grounds for a downward spiral. Through a longitudinal approach, identification of factors that over time influence the association between chronic stress and subsequent psychopathology may help to find those most at risk of a detrimental outcome. This study aimed to contribute to the current knowledge base by examining simultaneously two moderators of the chronic stress- psychopathology relationship: Basal cortisol and parental psychiatric history, hypothesizing that the moderator effect of basal cortisol will manifest especially in vulnerable individuals as indexed by parental psychiatric history.

Cortisol, the hormonal end product of the hypothalamic–pituitary–adrenal axis (Hawes et al. 2009) is increasingly of interest as a potential factor in the pathogenesis of externalizing and internalizing problems. Basal cortisol activity refers to cortisol levels in the absence of acute stress (Alink et al. 2008). Recent evidence suggests that as much as 41–57 % of variance in basal cortisol is accounted for by trait factors, that is, stable across days (Kertes and Van Dulmen 2012), and even that basal cortisol may be more informative with respect to trait characteristics than stress-induced cortisol reactivity (Laceulle et al. 2014). Still, basal cortisol has received relatively little attention. Reported associations of atypical basal cortisol levels with psychopathology have been inconsistent and typically weak (Alink et al. 2008; Knorr et al. 2010; Lopez-Duran et al. 2009; Ruttle et al. 2011). Within our research group, earlier analyses of the relationship between basal cortisol and psychopathology yielded similar weak and inconsistent findings (Dietrich et al. 2013; Hartman et al. 2013).

We propose that environmental influences may explain some of these inconsistencies and mask potentially robust associations, as basal cortisol has been related to context sensitivity (Ostiguy et al. 2011; Shirtcliff and Essex 2008). High context sensitivity as indexed by high basal cortisol may act as a risk for psychopathology following environmental instability (e.g., chronic stress), while low context sensitivity as indexed by low basal cortisol may be protective (Shirtcliff and Essex 2008).

We further propose that individual differences in vulnerability may explain additional variance in mental health outcome following chronic stress. According to the diathesis-stress model (Zuckerman 1999), effects of environmental stressors may primarily occur in vulnerable individuals. An important general vulnerability factor is parental psychiatric history, which is considered a strong predictor of increased genetic and environmentally-driven vulnerability for externalizing and internalizing psychopathology in offspring (Burke et al. 2002; Kim-Cohen et al. 2005).

We tested whether the combination of high awakening cortisol level at age 11 and the presence of a parental psychiatric history disproportionally increases adolescents’ risk of externalizing and internalizing problems following chronic stress. Adolescence is an important developmental period to study because of its high incidence of psychopathology. We hypothesized that individuals with high basal cortisol, assumed in the literature to reflect high context sensitivity, would be especially prone to these mental health problems following chronic stress in the presence of a parental psychiatric history, and less prone in its absence. We expected low basal cortisol, assumed to reflect low context sensitivity, to protect against the impact of chronic stress on mental health, even in the presence of a parental psychiatric history.

## Method and Materials

### Sample

We derived the data used in this study from the longitudinal “TRacking Adolescents’ Individual Lives Survey.” TRAILS aims to contribute to the understanding of the etiology of mental health problems by following 10- to 12-year-old Dutch children biennially into adulthood (Huisman et al. 2008; Ormel et al. 2012).

To obtain a large sample with wide ranges of parental psychiatric history severity, problem severity, and chronic stress, we pooled data from the TRAILS population-based birth cohort (*n* = 2230) and the parallel clinic-referred cohort (*n* = 543). We included three measurement waves: T1, T2, and T3, with mean ages about 11, 13.5, and 16 years, respectively. The sampling procedures, descriptive statistics, and response rates of both cohorts are well-documented (e.g., De Winter et al. 2005; Huisman et al. 2008; Ormel et al. 2012). In brief, TRAILS approached 135 primary schools in five municipalities in the Northern Netherlands to build the population cohort. Of these schools, 90.4 % agreed to participate. TRAILS contacted eligible students and their parents, enrolling 76 % (*n* = 2230) of those contacted in the study. The three data waves we included in this study ran from March 2001 to July 2002 (T1), September 2003 to December 2004 (T2), and September 2005 to August 2007 (T3), with response rates consistently above 80 %. The smaller, clinic-referred sample (*n* = 543) consisted of preadolescents who had been referred to the Groningen University Child and Adolescent Psychiatric Outpatient Clinic at any point in their life (20.8 % ≤5 years, 66.1 % 6–9 years, 13.1 % 10–12 years) for consultation or treatment. The first three data waves in the clinic-referred cohort ran 2 years behind those of the population cohort: From September 2004 to December 2005 (T1), September 2006 to November 2007 (T2), and September 2009 to February 2011 (T3). The measurement instruments and design for the clinic-referred cohort were the same as those of the population cohort. Of the 1264 eligible preadolescents, 543 (65.9 % boys; mean age 11.11; standard deviation [SD] 0.50; range 10.13–12.40) enrolled in the study and finished baseline measurements (T1). Of these 543 baseline participants, 85.1 % (*n* = 462) participated in the second wave (T2). Of the T2 participants, 83.5 % (*n* = 386) also participated in the third wave (T3). Another 30 T2 dropouts agreed to participate in the third wave, resulting in a total T3 response rate of 76.6 % (*n* = 416) of the original sample. Selective attrition analyses have been described elsewhere (De Winter et al. 2005; Huisman et al. 2008; Nederhof et al. 2012b; Ormel et al. 2012). Importantly, baseline participants did not differ from nonparticipants with respect to internalizing or externalizing problems. However, boys, preadolescents with a lower socioeconomic background and those with poor school performance were less likely to participate. At follow-up, small but significant differences existed between participants and nonparticipants in that attrition was somewhat more likely in boys and in adolescents with a nonwestern ethnicity, divorced parents, low socio-economic background, low peer status, low IQ, low academic achievement, poor physical health, or externalizing problems (De Winter et al. 2005; Huisman et al. 2008; Ormel et al. 2012). Nederhof et al. (2012b) showed that extensive recruitment effort increased representativeness of the TRAILS sample, although attrition was still selective.

Parents gave written informed consent prior to each assessment wave. Adolescents gave written informed assent at the second and third waves. TRAILS was approved by the National Dutch Medical Ethics Committee.

### Measures

#### Externalizing and Internalizing Problems

TRAILS used the Achenbach System of Empirically Based Assessment (ASEBA) family of measures of externalizing and internalizing problems (Achenbach and Rescorla 2001; Verhulst and Van der Ende 2013) at each time point. The Child Behavior Checklist is the parent-report questionnaire that contains 120 items assessing behavioral and emotional problems in children over the past 6 months. These items can be rated as 0 = *not true*, 1 = *somewhat or sometimes true*, or 2 = *very or often true*. We used DSM-IV-oriented subscales to define externalizing problems as the sum of the average scores of oppositional deviant problems, *k* = 5, Cronbach’s α = 0.80, and conduct problems, *k* = 17, α = 0.80, and internalizing problems as the sum of average anxiety, *k* = 6, α = 0.71, and affective problems, *k* = 13, α = 0.70. We repeated this procedure for the DSM-oriented subscales of the Youth Self-Report (Achenbach and Rescorla 2001). Again, externalizing problems were defined as the summed average of oppositional deviant, *k* = 5, α = 0.65, and conduct problems, *k* = 15, α = 0.73, and internalizing problems as the summed average of anxiety, *k* = 6, α = 0.61, and affective problems, *k* = 13, α = 0.71. We chose to use average scale scores and add the results in order to balance the influence of subscales with different numbers of items, then standardized the sum scores.

Externalizing problems correlated significantly, *p* < 0.001, with internalizing problems, *r* = 0.50 at T2 and *r* = 0.52 at T3 for parent-report and *r* = 0.40 at T2 and *r* = 0.33 at T3 for self-report. Due to this overlap, our main focus was on residual externalizing and internalizing scores, specifically, externalizing problems adjusted for co-occurring internalizing problems and vice versa. In addition, we used total problems, which include the shared variance between internalizing and externalizing problems. Results on unadjusted externalizing and internalizing are nonetheless described.

#### Chronic Stress Preceding T2 and T3

We operationalized chronic stress at T2 and T3 as the number of parent-reported long-term difficulties since the previous measurement. One of the parents, typically the mother, filled out a questionnaire that listed long-term difficulties to which the adolescent might have been exposed since the previous interview. The stressors included: (1) chronic illnesses or physical handicaps of the child (2) or a family member; (3) high work pressure at school; (4) housing problems; (5) neighborhood problems, such as violence or discrimination; (6) financial problems; (7) lack of friends; (8) being bullied; (9) long-lasting conflicts with family members (10) or others; and (11) long-lasting conflicts between family members. On an open item, parents could also disclose additional long-term difficulties. We coded these additional problems either as a long-term difficulty or dismissed them according to well-defined rules: In particular whether the described situation is typically considered stressful and enduring. For example, we coded a turbulent home environment, such as moving frequently from house to house or parents having an on/off relationship, as long-term difficulty. Situations that we rejected as long-term difficulty included normative or non-enduring situations such as the transition to middle school, puberty, and quarrels with siblings. The number of reported difficulties ranged from 0 to 10. To reduce the influence of extreme and rare scores, we grouped subjects into 4 categories; 0, 1, 2, or 3 or more long-term difficulties.

#### Cortisol Measurement

At T1, participants received verbal and written instructions to collect saliva at home on a normal school day without any stressful or special events, and in the absence of menstruation, feelings of illness, and (if possible) medication. Participants collected saliva upon waking while still in bed (Cort1) and 30 min later (Cort2), using the Sarstedt Salivette device (Nümbrecht, Germany). This device consists of a plastic sampling vessel with a suspended insert containing a sterile neutral cotton wool swab that has to be chewed for about 45 s and then returned to the insert (Rosmalen et al. 2005). The instructions stated that participants should rinse their mouth thoroughly with tap water and refrain from brushing their teeth, eating, or drinking (other than water) until after the second sample was collected. Any deviations from the protocol were reported on an accompanying form. Participants mailed the samples as soon as possible and kept them in the freezer prior to that. At the institute, samples were kept frozen (−20 °C) until analysis (Dietrich et al. 2013). Salivary cortisol (nmol/L) was measured by radioimmunoassay, with intra- and interassay coefficients of variation of 4.0 to 8.2 % and 5.6 to 12.6 %, respectively (Dietrich et al. 2013). A detailed description of the determination of cortisol levels is available elsewhere (Rosmalen et al. 2005), as well as information regarding the availability of cortisol samples and reasons for nonresponse in the population cohort (Rosmalen et al. 2005) and the clinic-referred cohort (Dietrich et al. 2013).

The current study focused on awakening cortisol level as an index of basal cortisol. Compared to other sampling times across the day, cortisol assessment immediately upon waking is less likely influenced by confounding factors such as food or caffeine consumption and physical activity level. We excluded all samples that were not collected within 10 min after waking to avoid influence of the cortisol awakening response (CAR), the typically steep increase in cortisol approximately 30 min after waking. The CAR has been suggested, because of its moderate heritability compared to basal cortisol, to primarily reflect state rather than trait characteristics (Laceulle et al. 2014).

#### Parental Psychiatric History

TRAILS assessed parent-reported family history at two time points using the TRAILS Family History Interview (Ormel et al. 2005). Prevalence rates using this instrument were comparable to reported rates from the large NEMESIS study, which were obtained using the Composite International Diagnostic Interview (Bijl et al. 1998; Ormel et al. 2005).

At T1, trained assistants conducted the interview. The second time (at T2 for the clinic-referred cohort, at T3 for the population cohort) parents filled out the interview themselves. After reading vignettes describing DSM-IV key symptoms for depression, anxiety, substance dependence, and persistent antisocial behavior, one parent was asked to indicate whether the behavior described in each vignette had ever applied to her/him and the other biological parent. We identified parents who indicated a definite episode (in contrast to a possible episode), reported in at least one of the two assessments, who in addition indicated having received treatment or medication during that episode, or, in case of antisocial behavior, had been in contact with the police.

Parental externalizing and internalizing problems are associated with both problem types in offspring (Marmorstein et al. 2004), suggesting that familial transmission (partly) results in a nonspecific vulnerability for psychopathology. Offspring’s vulnerability is further increased when a parent is affected by both types of psychopathology (Kim-Cohen et al. 2005), whereas offspring of two affected parents may be at even higher risk for psychopathology (Dierker et al. 1999; Marmorstein et al. 2004). We therefore indexed the severity of parental psychiatric history, hereafter referred to as parental history (PH), for each adolescent by counting the number of definite diagnosis dimensions in the parents, resulting in four groups: 0 = *no psychiatric history in either parent*; 1 = *one parent with either externalizing or internalizing history*; 2 = *one parent with both externalizing and internalizing history* or *two parents with either externalizing or internalizing history*; 3 = *one parent with both externalizing and internalizing history and either externalizing or internalizing history in the other parent,* or *externalizing and internalizing history in both parents*. We labeled the groups as follows: 0 = *no PH*; 1 = *mild PH*; 2 = *severe PH*; and 3 = *very severe PH*.

#### Covariates

We corrected for a number of possibly confounding factors. Covariates included age, sex (0 = *female*, 1 = *male*), a quadratic correction factor for cortisol sampling month (Rosmalen et al. 2005), and preadolescents’ medication use as reported by their parents. We categorized medication use into four dichotomous variables (0 = *no*, 1 = *yes*); corticosteroids (*n* = 22), methylphenidate (*n* = 205), other psychotropic medication (e.g., antidepressants, antipsychotics, *n* = 58), and other somatic medication (*n* = 74).

### Data Analysis

#### Data Preparation and Preliminary Analyses

In the case of noncompliance (i.e., not within 10 min of waking) or when information on sampling time was ambiguous or missing, we regarded awakening cortisol levels as invalid. We changed extreme values (more than three SDs from the mean) and invalid cases to missing. In case these participants did have a valid value on the second cortisol sample, collected 30 minutes after waking, we imputed awakening cortisol levels on the basis of the first assay’s group mean plus the participant’s standardized value of the second assay times the first assay’s SD, in line with previous studies (Booij et al. 2013; Bouma et al. 2009; Hartman et al. 2013). A posthoc check showed that results did not change if we deleted individuals who had imputed cortisol data.

For this study, our statistical analysis method required at least one value for each predictor on T1-T3 and at least T2 or T3 externalizing and internalizing problems. Thus, we needed complete externalizing and internalizing psychiatric history of both parents, reported in at least one of the two assessments, to reliably compute parental psychiatric history severity, and needed T1 basal cortisol, T2 and/or T3 chronic stress, and T2 and/or T3 parent-reported and/or self-reported externalizing and internalizing problems. We performed independent samples *t*-tests to check whether included and excluded subjects differed with respect to our study variables and to determine which medication groups influenced basal cortisol and would thus have to be included as a covariate in our main analyses.

#### Main Analyses

We computed Pearson’s correlation coefficients between the predictors and T2 and T3 parent-reported and self-reported externalizing and internalizing problems. We used Linear Mixed Modeling (LMM) to predict T2 and T3 problem levels from parental history severity, basal cortisol, chronic stress, and their interactions. LMM allows for missing data at different measurement waves, which is an important advantage for a longitudinal design (Kwok et al. 2008). Using PASW Statistics 18, we conducted LMM analyses (T2 and T3 in a single analysis) separately for total problems, adjusted externalizing problems (EXTadj), and adjusted internalizing problems (INTadj). We conducted separate analyses for parent-reported and self-reported data on mental health.

We included the independent variables of age (time-variant covariate), sex, cortisol sampling month correction, influential medication groups, chronic stress (time-variant), basal cortisol, parental history severity, and all possible interactions between chronic stress, basal cortisol, and parental history severity. We allowed for a curvilinear (quadratic) effect in basal cortisol, since basal cortisol may predict problem level especially through extremes in the basal cortisol distribution, rather than linearly. We used the Maximum Likelihood estimation procedure and considered a *p*-value < 0.05 to be statistically significant.

For interpretation of interaction effects, we plotted the outcome variable based on the estimated regression coefficients, for different levels of each predictor. For every significant three-way interaction effect of basal cortisol, chronic stress, and parental history, we tested to what extent the effect applied to extremes in the parental history severity distribution. We did so by testing whether an interaction effect of basal cortisol and chronic stress was significant for very severe PH, for no PH, or both. We computed estimates, standard errors, and *p*-values using SAS 9.3.

To investigate to what extent pre-existing mental health problems account for our findings, we repeated analyses with initial problem levels at T1 as an additional covariate. Specifically, we conducted the analyses of total problems with T1 total problems as a covariate, the analyses of EXTadj with T1 EXTadj, and the analyses of INTadj with T1 INTadj.

To check the influence of adjusting externalizing and internalizing problems for their co-occurrence, we performed additional analyses with unadjusted externalizing problems (EXT) and unadjusted internalizing problems (INT).

## Results

### Results of Preliminary Analyses

Four hundred seventy-eight adolescents (*n* = 460 population cohort, *n* = 18 clinic-referred cohort) did not participate in the saliva (cortisol) collection. Of those who did participate, cortisol measurement failed in 155 cases, whereas cortisol values were invalid in 57 cases (*n* = 32 noncompliance, *n* = 25 extreme values). We imputed basal cortisol values of 111 subjects, which resulted in a total of 2194 valid basal cortisol values (*n* = 1753 population cohort, *n* = 441 clinic-referred cohort) and 579 missing or invalid values (*n* = 477 population cohort, *n* = 102 clinic-referred cohort). Three hundred and nine participants had missing data for both measurements of chronic stress, 218 for parental history, and 137 for parent-reported as well as self-reported externalizing and internalizing problems. Altogether, we excluded a total of 856 participants (30.9 % of the original sample; 78.5 % population cohort) from this study, resulting in a final sample size of 1917 subjects (69.1 % of the original sample; mean age 11.09; SD 0.54; range 10.01–12.58; 53.2 % boys; 81.3 % population cohort). Compared to excluded individuals, included subjects were slightly younger, *t* (1566.18) = −2.89, *p* < 0.01, had less chronic stress between T1 and T2, *t* (715.19) = −3.48, *p* < 0.001, and fewer parent-reported externalizing problems, *t* (1168.36) = −3.61, *p* < 0.001, and internalizing problems, *t* (1174.45) = −2.43, *p* < 0.05. The groups did not differ with respect to sex, PH severity, basal cortisol, and self-reported externalizing and internalizing problems.

Comparison of basal cortisol levels of 1 = *users* and 0 = *non*-*users* of the four medication groups showed significantly lower basal cortisol levels in users of methylphenidate, *t* (239.92) = 11.86, *p* < 0.001, and users of other psychotropics, such as antipsychotics or antidepressants, *t* (51.97) = 4.36, *p* < 0.001. Basal cortisol levels were not significantly associated with corticosteroids, *p* = 0.98, or other somatic medication, *p* = 0.45. Based on these results we included use of methylphenidate and other psychotropics (in the final sample: *n* = 149 and *n* = 37, respectively) as covariates.

### Descriptive Statistics and Correlations

Table 1 shows descriptive statistics of the final sample and Table 2 correlations between predictors and adjusted parent-reported and self-reported externalizing and internalizing problems. Chronic stress correlated significantly, *p* < 0.001, with total problems reported by parents at T2 and T3, *r* = 0.50 and *r* = 0.48, respectively, and adolescents, *r* = 0.23 and *r* = 0.25, respectively.

**Table 1:** Descriptive statistics of the variables used in this study

Variable		*N*	Mean (SD)	Range	*N*	Mean (SD)	Range
Age	T1	1916	11.09 (0.54)	10.01–12.58			
	T2	1896	13.41 (0.59)	11.58–15.03			
	T3	1672	16.17 (0.68)	14.42–18.48			
Stress^a^	T2	1867	1.16 (1.44)	0–10			
	T3	1541	1.27 (1.53)	0–10			
Cortisol	T1	1917	10.87 (4.73)	0.71–34.99			
PH severity	0	1061					
	1	632					
	2	185					
	3	39					
		Parent-report			Self-report		
EXT^b^	T1	1873	5.83 (4.86)	0–33	1889	5.90 (4.30)	0–28
	T2	1866	4.41 (4.60)	0–31	1849	5.75 (4.14)	0–29
	T3	1532	4.25 (4.77)	0–28	1556	5.96 (4.41)	0–24
INT^c^	T1	1873	5.13 (4.47)	0–27	1889	6.09 (4.54)	0–28
	T2	1866	3.92 (4.16)	0–24	1849	5.76 (4.65)	0–33
	T3	1532	3.66 (4.09)	0–29	1556	5.80 (4.79)	0–35
TOT^d^	T1	1873	10.96 (8.02)	0–50	1889	11.99 (7.57)	0–56
	T2	1866	8.33 (7.63)	0–46	1849	11.51 (7.33)	0–47
	T3	1532	7.91 (7.78)	0–49	1556	11.76 (7.47)	0–45

*Cortisol* Awakening cortisol level, *PH* Parental history, *EXT* Externalizing problems, *INT* Internalizing problems, *TOT* Total problems

^a^Number of long-term difficulties experienced since previous measurement

^b^Sum of scores on 22 items for parent-report and 20 items for self-report

^c^Sum of scores on 19 items for both parent-report and self-report

^d^Sum of externalizing and internalizing problems

**Table 2:** Correlation matrix of predictors and outcome variables, with parent-reported mental health problems below and self-reported problems above diagonal

						Self-report			
	Variable	T2Stress	T3Stress	Cortisol	PH	T2EXTadj	T3EXTadj	T2INTadj	T3INTadj
	T2Stress	1	0.56***	−0.09***	0.17***	0.07**	0.05*	0.18***	0.18***
	T3Stress	0.56***	1	−0.09***	0.24***	0.06*	0.08**	0.20***	0.20***
	Cortisol	−0.09***	−0.09***	1	−0.03	−0.04	−0.06*	0.01	0.04
	PH	0.17***	0.24***	−0.03	1	0.04	0.02	0.07**	0.06*
Parent-report	T2EXTadj	0.16***	0.13***	−0.05*	0.11***	0.40***	0.45***	−0.40***	−0.14***
	T3EXTadj	0.11***	0.11***	−0.05	0.05	0.62***	0.44***	−0.12***	−0.33***
	T2INTadj	0.36***	0.27***	−0.04	0.12***	−0.50***	−0.27***	0.36***	0.49***
	T3INTadj	0.26***	0.38***	−0.02	0.17***	−0.25***	−0.51***	0.56***	0.44***

*Cortisol* Awakening cortisol level, *PH* Parental history severity, *EXTadj* Externalizing problems adjusted for internalizing problems, *INTadj* Internalizing problems adjusted for externalizing problems

***p < 0.001, ** *p* < 0.01, * *p* < 0.05

### Parental History Severity, Basal Cortisol, and Chronic Stress

Parent-reported and self-reported total problems were not significantly predicted by a three-way interaction of parental history severity, squared or linear basal cortisol, and chronic stress, *p*-values ranged from 0.21 to 0.97. Interaction effects remained statistically nonsignificant after correcting for initial total problems at T1, *p*-values ranged from 0.12 to 0.71.

**Fig. 1:**
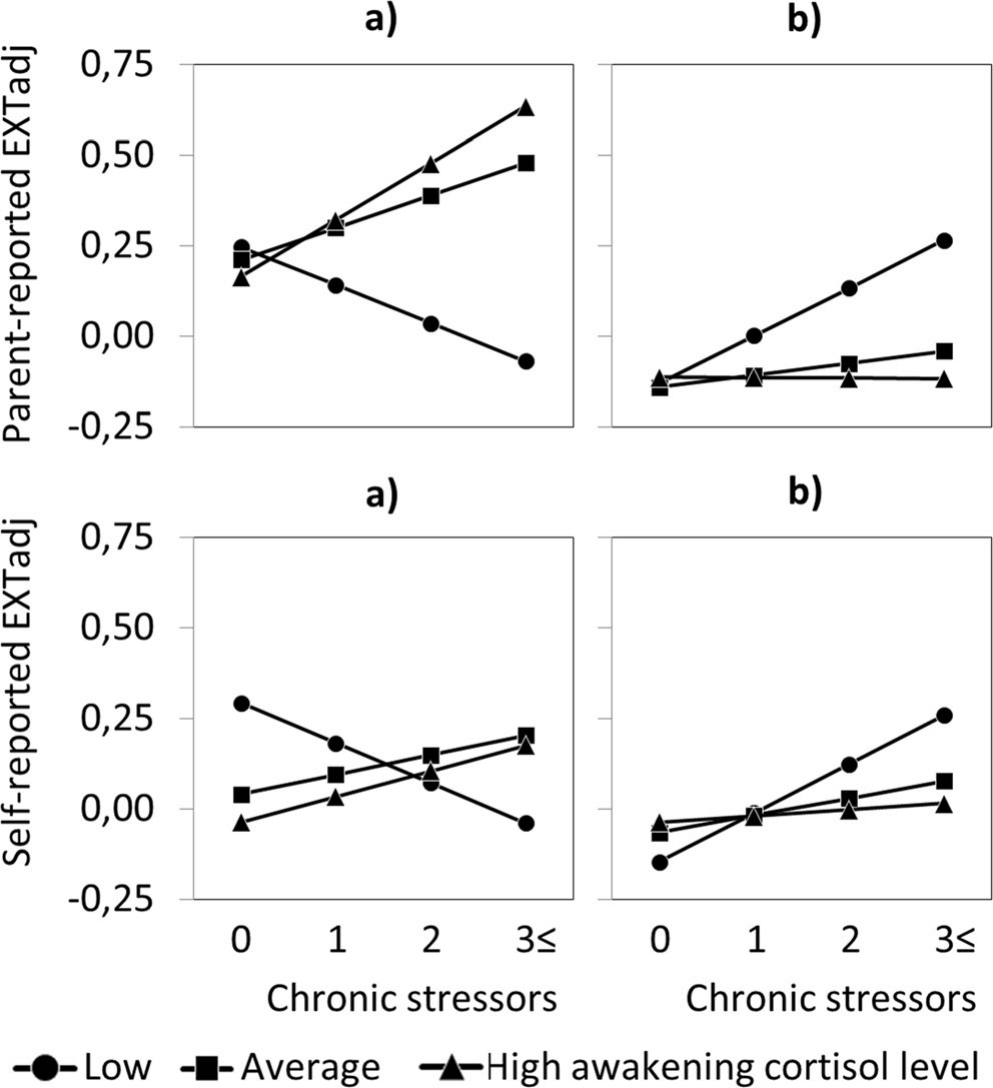
Parent-reported (*upper panel*) and self-reported (*lower panel*) externalizing problem levels plotted for different levels of chronic stress and basal cortisol, and separately depicted for very severe PH (**a**) and no PH (**b**). *Note. PH* Parental history severity, *EXTadj* Externalizing problems adjusted for internalizing problems. Levels of chronic stress refer to the number of long-term difficulties at T2. Low, average, and high cortisol (−1SD, M, and +1SD) correspond to 6.15, 10.87, and 15.60 nmol/L, respectively

We did however find significant interaction effects in predicting EXTadj as well as INTadj problems. Both parent-reported and self-reported EXTadj problems were significantly predicted by a three-way interaction of PH severity, squared basal cortisol, and chronic stress, *p* = 0.018 and 0.019, respectively (Table 3). We plotted EXTadj levels for low, average, high, and very high levels of the truncated chronic stress variable (corresponding to 0, 1, 2, and 3 or more long-term difficulties at T2, respectively) and low, average, and high basal cortisol (−SD, M, and +1SD, corresponding to 6.15, 10.87, and 15.60 nmol/L), separately for the two extremes of the PH severity distribution; very severe PH versus no PH. Figure 1 shows comparable patterns for parent-reported EXTadj (upper panel) and self-reported EXTadj (lower panel). In adolescents with very severe PH (a) low basal cortisol attenuated the association between chronic stress and EXTadj levels, whereas high basal cortisol somewhat enhanced the association between chronic stress and parent-reported but not self-reported EXTadj levels.

**Table 3:** Parental history severity, squared basal cortisol, and chronic stress significantly predicted parent-reported and self-reported externalizing problems

	Parent-reported EXTadj			Self-reported EXTadj		
Parameter	Estimate^a^	SE^a^	*p*	Estimate^a^	SE^a^	*p*
Intercept^b^	−303.19	196.27	0.12	−433.36	212.35	0.04
Age	8.99	7.74	0.25	−4.97	9.32	0.59
Sex^c^	153.43	41.53	<0.001	282.34	40.67	<0.001
Sampling month	−111.82	68.49	0.10	−41.99	66.95	0.53
Methylphenidate	421.09	80.95	<0.001	288.45	79.27	<0.001
Other psychotropics	444.39	153.05	0.004	−117.58	148.44	0.43
Stress	358.85	95.22	<0.001	334.87	103.71	0.001
Cortisol	−16.86	26.46	0.52	37.15	27.04	0.17
Cortisol^2^	0.87	1.01	0.39	−1.18	1.03	0.25
PH	112.30	183.94	0.54	404.51	188.09	0.03
Cortisol*Stress	−45.73	16.39	0.005	−40.40	17.83	0.02
Cortisol^2^*Stress	1.45	0.64	0.02	1.28	0.70	0.07
PH*Cortisol	4.53	29.59	0.88	−52.43	30.23	0.08
PH*Cortisol^2^	−0.37	1.09	0.73	1.70	1.11	0.12
PH*Stress	−302.38	95.33	0.002	−294.11	103.15	0.004
PH*Cortisol*Stress	45.11	15.95	0.005	43.96	17.27	0.011
PH*Cortisol^2^*Stress	−1.43	0.60	0.018	−1.54	0.65	0.019

*Cortisol* Awakening cortisol level, *Cortisol* ^*2*^ Squared awakening cortisol level, *PH* Parental history severity, *EXTadj* Externalizing problems adjusted for internalizing problems

^a^Values multiplied by 1000 for increased readability

^b^Participants varied significantly, *p* < 0.01, in intercept for parent-reported EXTadj, var(*u*0*j*) = 560.84^a^, chi-square(1) = 633.76, and self-reported EXTadj, var(*u*0*j*) = 413.74^a^, chi-square(1) = 281.79

^c^Sex was coded as 0 = *female*, 1 = *male*

It appeared as if low basal cortisol adolescents with very severe PH had lower levels of EXTadj under high chronic stress than under low chronic stress. However, posthoc estimate and standard error calculation for the slope of EXTadj across chronic stress levels showed that it did not significantly differ from zero, for parent-report and self-report, *p* = 0.48 and *p* = 0.14, respectively, and should thus be interpreted as stable across chronic stress levels.

Given no PH (b), low basal cortisol did not attenuate but rather enhanced the association between chronic stress and parent-reported and self-reported EXTadj levels, *p*-values of the stress slope < 0.001. Chronic stress had no impact on EXTadj levels in no PH-adolescents with average or high basal cortisol.

**Fig. 2:**
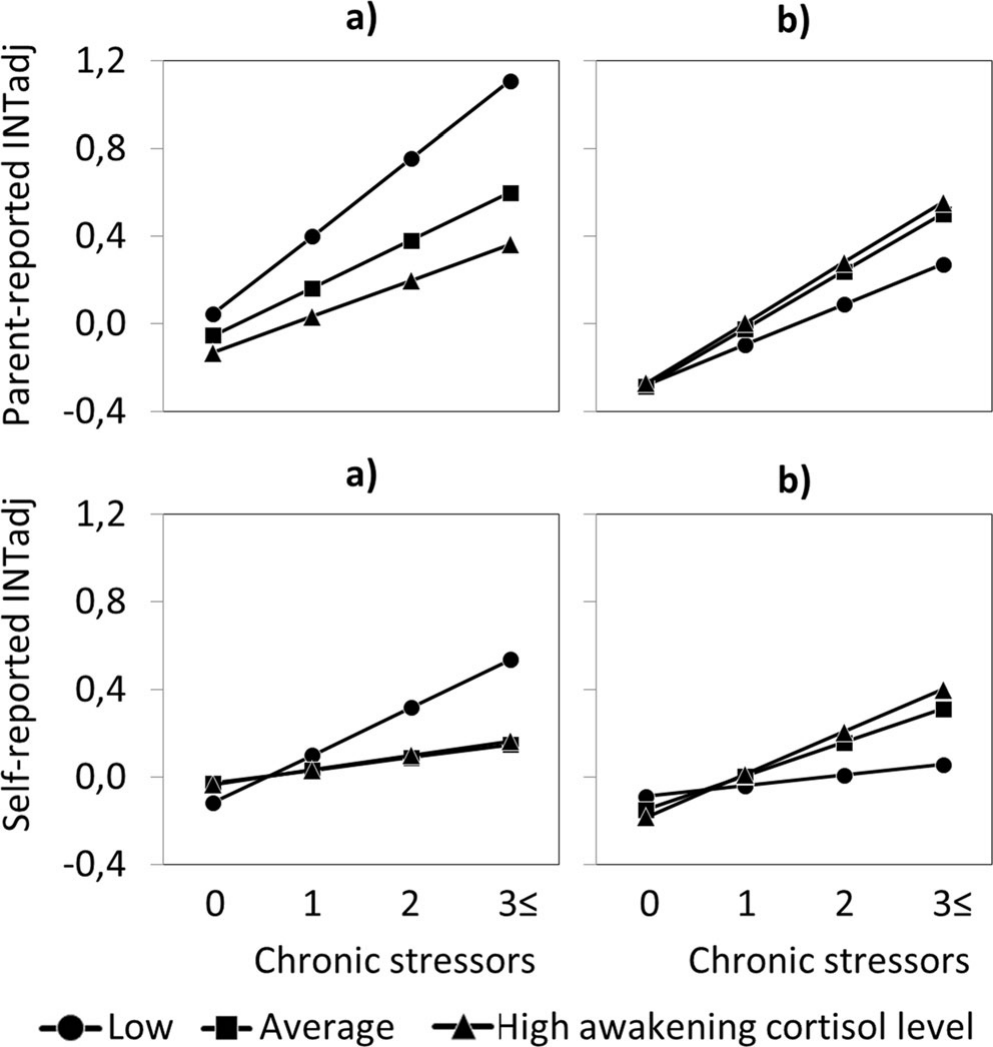
Parent-reported (*upper panel*) and self-reported (*lower panel*) internalizing problem levels plotted for different levels of chronic stress and basal cortisol, and separately depicted for very severe PH (**a**) and no PH (**b**). Note. *PH* Parental history severity; *INTadj* Internalizing problems adjusted for externalizing problems. Levels of chronic stress refer to the number of long-term difficulties at T2. Low, average, and high cortisol (−1SD, M, and +1SD) correspond to 6.15, 10.87, and 15.60 nmol/L, respectively

INT_adj_ problems were predicted by a three-way interaction of PH severity, squared basal cortisol, and chronic stress, which was marginally significant for parent-report, *p* = 0.06, and significant for self-report, *p* = 0.006, as shown in Table 4. Figure 2 shows that the pattern in which parent-reported (upper panel) and self-reported (lower panel) INTadj levels were predicted is opposite to that found for parent-reported and self-reported EXTadj levels. That is, the association between chronic stress and subsequent INT_adj_ levels was enhanced in individuals with low basal cortisol and very severe PH (a) and attenuated in individuals with low basal cortisol and no PH (b). Average and high basal cortisol did not differ substantially with respect to stress impact on INTadj level.

**Table 4:** Parental history severity, squared basal cortisol, and chronic stress significantly predicted parent-reported and self-reported internalizing problems

	Parent-reported INTadj			Self-reported INTadj		
Parameter	Estimate^a^	SE^a^	*p*	Estimate^a^	SE^a^	*p*
Intercept^b^	21.29	193.69	0.91	611.93	202.88	0.003
Age	−15.98	8.11	0.05	−22.25	8.95	0.013
Sex^c^	−141.78	38.87	<0.001	−542.28	38.64	<0.001
Sampling month	−0.77	64.09	0.99	34.66	63.61	0.59
Methylphenidate	154.45	75.84	0.04	−13.59	75.33	0.86
Other psychotropics	658.83	143.53	<0.001	202.30	141.07	0.15
Stress	−17.20	94.28	0.86	−181.67	99.00	0.07
Cortisol	−8.94	25.32	0.72	−22.74	25.74	0.38
Cortisol^2^	0.45	0.97	0.64	0.58	0.98	0.55
PH	148.86	176.93	0.40	−136.61	179.15	0.45
Cortisol*Stress	41.84	16.23	0.010	46.12	17.02	0.007
Cortisol^2^*Stress	−1.48	0.64	0.02	−1.41	0.67	0.03
PH*Cortisol	−6.70	28.45	0.81	26.28	28.80	0.36
PH*Cortisol^2^	0.01	1.04	0.99	−0.92	1.05	0.38
PH*Stress	225.63	94.27	0.017	285.21	98.45	0.004
PH*Cortisol*Stress	−34.34	15.80	0.03	−47.79	16.49	0.004
PH*Cortisol^2^*Stress	1.12	0.60	0.06	1.72	0.62	0.006

*Cortisol* Awakening cortisol level, *Cortisol* ^*2*^ Squared awakening cortisol level, *PH* Parental history severity, *INTadj* Internalizing problems adjusted for externalizing problems

^a^Values multiplied by 1000 for increased readability

^b^Participants varied significantly, *p* < 0.01, in intercept for parent-reported INTadj, var(*u*0*j*) = 436.52^a^, chi-square(1) = 418.68, and self-reported INTadj, var(*u*0*j*) = 366.65^a^, chi-square(1) = 268.38

^c^Sex was coded as 0 = *female*, 1 = *male*

Posthoc, we tested to what extent the aforementioned three-way interaction effects of squared basal cortisol, chronic stress, and PH applied to the extremes of the PH severity distribution (two-way interaction effect estimates and standard errors are available upon request). Given a very severe PH, a two-way interaction effect of squared basal cortisol and chronic stress was statistically significant in predicting parent-reported EXTadj, *p* = 0.044, and self-reported EXTadj, *p* = 0.030. Given no PH, the two-way interaction effect was significant in predicting parent-reported EXTadj, *p* = 0.024, but was weaker in predicting self-reported EXTadj, *p* = 0.07. An interaction effect of chronic stress with linear rather than squared basal cortisol was significant given very severe PH, *p* = 0.018 and *p* = 0.026 for parent-report and self-report, respectively, and given no PH, *p* = 0.005 and *p* = 0.023 for parent-report and self-report, respectively. Thus, the three-way interaction effect that was significant in predicting parent-reported and self-reported EXTadj applied to both extremes of the PH severity distribution.

Given a very severe PH, a two-way interaction effect of squared basal cortisol and chronic stress was not significant in predicting parent-reported INTadj, *p* = 0.18, but significantly predicted self-reported INTadj, *p* = 0.011. Given no PH, the two-way interaction effect was significant in predicting parent-reported as well as self-reported INTadj, *p* = 0.020 and *p* = 0.034, respectively. An interaction effect of chronic stress with linear rather than squared basal cortisol approached marginal significance in predicting parent-reported INTadj given a very severe PH, *p* = 0.10, and was significant in predicting self-reported INTadj given a very severe PH, *p* = 0.013, as well as parent-reported and self-reported INTadj given no PH, *p* = 0.010 and *p* = 0.007, respectively. Thus, the three-way interaction effect that was marginally significant in predicting parent-reported INTadj applied more to no PH than to very severe PH, whereas the significant three-way interaction effect in predicting self-reported INTadj applied to both extremes of the PH severity distribution. Note that a very severe PH is much less common and has therefore less power, which may in part explain lower *p*-values compared to no PH.

We performed additional analyses of externalizing and internalizing problems adjusted for their co-occurrence, while correcting for initial problem levels at T1 (for tables and figures see Online Resource 1). Results were in the same direction but weaker, which is consistent with the expectation that stress and FH severity already partly exerted their effects before T1. We also performed additional analyses of externalizing and internalizing problems unadjusted for their co-occurrence (for tables and figures see Online Resource 2). Results were in the same direction, but less outspoken, consistent with diverging effects for externalizing and internalizing problems that get evened out with the inclusion of the variance shared by both. On both extremes of the PH distribution and across informants, adolescents with high basal cortisol did not differ substantially from those with average basal cortisol with respect to EXTadj and INTadj levels adjusted for baseline problem level, nor with respect to externalizing and internalizing problems unadjusted for their co-occurrence. Therefore, though our data consistently show an effect of low basal cortisol on the stress- psychopathology relationship, they do not show an effect of high basal cortisol.

## Discussion

This study examined whether the combination of high basal cortisol and the presence of a parental psychiatric history disproportionally increases the risk of externalizing and internalizing problems following chronic stress from pre-adolescence into adolescence. In general, higher chronic stress exposure was associated with more externalizing and internalizing problems, consistent with the literature. In addition, we found a complex interaction of basal cortisol and parental psychiatric history with chronic stress in predicting subsequent risk of externalizing and internalizing problems adjusted for their overlap. We did not find a three-way interaction effect for total problems, possibly due to opposing interaction effects for externalizing versus internalizing problems.

In adolescents, low basal cortisol combined with the absence of a parental psychiatric history increased risk of externalizing but not internalizing problems following chronic stress. Conversely, low basal cortisol combined with a substantial parental psychiatric history increased risk of internalizing but not externalizing problems following chronic stress. Thus, parental psychiatric history moderated stress- cortisol interactions in predicting psychopathology, but in a different direction than hypothesized. Namely, (1) findings were opposite for externalizing and internalizing problems, and (2) effects pertained to low basal cortisol and not high basal cortisol.

Regarding the first, what conferred risk of externalizing problems following chronic stress protected against internalizing problems following chronic stress and vice versa. These opposite findings for externalizing and internalizing problems suggest that context sensitivity may not refer to a general trait. Rather, what confers context sensitivity may not only be dependent on the type of context (Nederhof et al. 2012a; Obradović et al. 2011) but also on the outcome of interest.

Regarding the second, we had hypothesized that individuals with high basal cortisol, an assumed indicator of high context sensitivity, would be especially prone to mental health problems following chronic stress in the presence of a severe parental psychiatric history, and less prone in the absence of parental psychiatric history. We did not find this. Rather, in all instances of high chronic stress, it was low basal cortisol that stood out from average or high basal cortisol with respect to psychopathology levels. Thus, our results suggest that an effect of basal cortisol on the stress- psychopathology relationship lies within the low cortisol range. This is not in line with the assumption that high basal cortisol constitutes high context sensitivity, on which we had based our hypotheses.

It should be noted in this context that we recently demonstrated, in line with the literature (Shirtcliff and Essex 2008), that high basal cortisol predicted a rise in mental health problems following the transition to middle school in adolescents who perceived the transition as negative (Zandstra et al. 2015). A plausible explanation for not finding an effect of high basal cortisol in the present study may be the focus on exposure to chronic stressors rather than a recent one-time stressor such as the transition to middle school. Research has shown that the experience of stress may initially cause increased activity of the stress system, but decreased activity over time (e.g., Ruttle et al. 2011). This is reffered to as down-regulation, a protective measure of the body against the negative health effects of excessive cortisol production. Decreased physiological stress sensitivity does not imply decreased psychological stress sensitivity (Fries et al. 2005; Gruenewald et al. 2006). Therefore, in individuals with low levels of mental health problems in the context of chronic stress, low basal cortisol may represent low context sensitivity, while in other, more context-sensitive individuals, basal cortisol may have been down-regulated following long-lasting stress exposure and may have been high in the past, in the early phase of stress exposure. Thus, individual differences in exposure to chronic stress may complicate the interpretation of basal cortisol as indicative of either high or low context sensitivity.

An alternative explanation may be that the association of high context sensitivity with high cortisol described in the literature relates more to cortisol reactivity and less to basal cortisol. In contrast, our findings may be true but appear unusual because of a publication bias in the basal cortisol literature. Future research should provide insight into the mechanisms at play.

The findings of this study should be interpreted in light of some limitations. First, we collected cortisol assays only once, inducing the possibility of a novelty effect and other state influences. Stable trait influences on cortisol will be more reliably assessed if morning cortisol is sampled for a number of consecutive days (Hellhammer et al. 2007). However, recent evidence suggests that single-day cortisol assessment is informative with respect to trait influences (Kertes and Van Dulmen 2012). Unfortunately, we lack the data to investigate whether individuals with high chronic stress levels and low basal cortisol may have had high basal cortisol in the past. Therefore, we cannot test our posthoc hypothesis of cortisol down-regulation following prolonged chronic stress exposure. Future research, conducting multiple cortisol assessments over time, may establish how individual cortisol levels are affected by recent-onset versus life-long chronic stress. A second limitation is the use of solely parent-reported data on chronic stress. The relatively high correlation between parent-reported chronic stress and parent-reported adolescent mental health problems is partially due to shared method variance. Although we analyzed both parent-reported and self-reported behavioral and emotional problems, self-reported data on chronic stress were not available. In adolescents with average or high cortisol, the association between parent-reported chronic stress and self-reported externalizing problems was rather weak and nearly absent after correcting for initial externalizing problems at T1. This pattern could reflect the tendency of adolescents to underreport their own externalizing behavior (Salbach-Andrae et al. 2009), for example, through denial or lack of insight. Alternatively, it may imply that stressors reported by parents were not that stressful for their offspring. Still, parents may be more reliable than their early adolescent offspring in assessing whether long-term situations are difficult or not (e.g., financial or housing problems). Third, the parental psychiatric history data do not reflect clinical diagnoses. Employing the gold standard, conducting comprehensive diagnostic interviews with both parents, was not possible due to financial and practical constraints, such as limited time and risk of increased dropout due to overdemanding research participants. We focussed on lifetime disorders that had ever involved a professional, such as a psychiatrist, a general practitioner, or law enforcement. Although this method enabled a reliable identification of individuals with a severe parental history of psychopathology, it may have led to underestimations in individuals with mild parental psychiatric problems. Furthermore, relying on one parent to describe the other parent’s psychiatric history may also have resulted in underestimations of participants’ parental history. Thus, potential bias in our parental history data may have led to underestimation of effects, rather than overestimation. Finally, individuals excluded from analyses based on missings had on average more parent-reported externalizing and internalizing problems and chronic stress than included individuals. Due to this attrition, estimates may be somewhat conservative.

Strengths of the study include the large sample of longitudinal, multi-informant data from preadolescence well into adolescence, large interindividual differences in externalizing and internalizing problem levels and chronic stress exposure, the use of Linear Mixed Modeling that allowed for optimal use of all available data from multiple measurements, and sensitivity analyses on prior problem severity.

In sum, we showed a complex interaction of basal cortisol and parental psychiatric history with chronic stress in predicting subsequent risk of externalizing and internalizing psychopathology that differed between these two major forms of psychopathology. Our findings may explain in part why reported associations between basal cortisol and psychopathology have been inconsistent and weak, including reports of TRAILS basal cortisol data (Alink et al. 2008; Dietrich et al. 2013; Hartman et al. 2013; Knorr et al. 2010; Lopez-Duran et al. 2009; Ruttle et al. 2011). When an effect of basal cortisol on psychopathology is conditional on other factors, in this case chronic stress levels and parental psychiatric history severity, it may be difficult to find a direct association (Moffitt et al. 2006). Our findings further suggest that the premise that basal cortisol reflects context sensitivity may be too crude. Individual differences in exposure to chronic stress may complicate the interpretation of basal cortisol as indicative of either high or low context sensitivity, since in some individuals low basal cortisol may be the result of down-regulation following long-lasting stress exposure. In addition, our results suggest that what confers context sensitivity may depend on the outcome of interest, as illustrated by opposite findings for externalizing and internalizing problems. Although consistent across informants, our findings need replication.

## Ethical Standards

TRAILS was approved by the National Dutch Medical Ethics Committee and has therefore been performed in accordance with the ethical standards laid down in the 1964 Declaration of Helsinki and its later amendments. Parents gave written informed consent prior to each assessment wave. Adolescents gave written informed assent at the second and third waves. This manuscript contains no information that discloses the identity of our participants or violates their privacy.

## Electronic supplementary material

Below is the link to the electronic supplementary material.

ESM 1 (DOCX 197 kb)

ESM 2 (DOCX 191 kb)
