# Supplementary material for: Chronic Stress and Adolescents’ Mental Health: Modifying Effects of Basal Cortisol and Parental Psychiatric History. The TRAILS Study
Source: J Abnorm Child Psychol. 2015 Jan 25;43(6):1119–30. doi: 10.1007/s10802-014-9970-x (PMC4494132; doi:10.1007/s10802-014-9970-x)
Supplement: Supplementary file 1 — (DOCX 197 kb) [file 10802_2014_9970_MOESM1_ESM.docx]

**Online Resource 1**

Tables and figures of EXTadj and INTadj corrected for initial problem level at T1.

This material is supplementary to:

Chronic stress and adolescents’ mental health: Modifying effects of basal cortisol and parental psychiatric history. The TRAILS study. *Journal of Abnormal Child Psychology.*

Anna Roos E. Zandstra, Catharina A. Hartman, Esther Nederhof, Edwin R. van den Heuvel, Andrea Dietrich, Pieter J. Hoekstra, Johan Ormel

Corresponding Author: Anna Roos Zandstra, Faculty of Mathematics and Natural Sciences, University of Groningen, The Netherlands. E-mail: A.R.E.Zandstra@rug.nl

**Table 1** Correcting for externalizing problems at baseline, a three-way interaction effect of parental history severity, squared basal cortisol and chronic stress significantly predicted parent-reported but not self-reported externalizing problems.

|  | Parent-reported EXTadj | | |  | Self-reported EXTadj | | |
| --- | --- | --- | --- | --- | --- | --- | --- |
| Parameter | Estimate*^a^* | SE*^a^* | *p* |  | Estimate*^a^* | SE*^a^* | *p* |
| Intercept*^b^* | -157.83 | 173.05 | .36 |  | -166.01 | 205.09 | .42 |
| Age | 10.17 | 7.63 | .18 |  | -7.37 | 9.26 | .43 |
| Sex*^c^* | -26.09 | 33.59 | .44 |  | 127.86 | 38.75 | **<.001** |
| Sampling month | -128.79 | 54.39 | **.018** |  | -51.50 | 62.41 | .41 |
| Methylphenidate | 150.32 | 64.57 | **.020** |  | 257.90 | 73.16 | **<.001** |
| Other psychotropics | -32.77 | 122.09 | .79 |  | -226.34 | 137.11 | .10 |
| T1 EXTadj | 582.91 | 17.08 | **<.001** |  | 345.53 | 19.09 | **<.001** |
| Stress | 293.85 | 83.80 | **<.001** |  | 238.91 | 99.08 | **.016** |
| Cort | -5.16 | 21.93 | .81 |  | 17.00 | 25.50 | .50 |
| Cort² | 0.24 | 0.84 | .78 |  | -0.58 | 0.97 | .55 |
| PH | -29.85 | 153.78 | .85 |  | 221.05 | 177.38 | .21 |
| Cort*Stress | -42.33 | 14.44 | **.003** |  | -25.47 | 17.04 | .14 |
| Cort²*Stress | 1.46 | 0.56 | **.010** |  | 0.80 | 0.67 | .23 |
| PH*Cort | 9.45 | 24.70 | .70 |  | -25.22 | 28.48 | .38 |
| PH*Cort² | -0.39 | 0.91 | .67 |  | 0.84 | 1.04 | .42 |
| PH*Stress | -219.19 | 83.55 | **.009** |  | -200.26 | 98.42 | **.042** |
| PH*Cort*Stress | 36.07 | 14.02 | **.010** |  | 28.08 | 16.49 | .09 |
| PH*Cort²*Stress | -1.23 | 0.53 | **.021** |  | -0.99 | 0.62 | .11 |

*Note.* Cort = Awakening cortisol level; Cort² = Squared awakening cortisol level; PH = Parental history severity; EXTadj = Externalizing problems adjusted for internalizing problems.

*^a^*Values multiplied by 1000 for increased readability.

*^b^*Participants varied significantly (*p<*.01) in intercept for parent-reported EXTadj, var(*u*0*j*)=266.64*^a^*, chi-square(1)=272.61, and self-reported EXTadj, var(*u*0*j*)=304.85*^a^*, chi-square(1)=188.32.

*^c^*Sex was coded as 0­ = *female*, 1 = *male*.

**Table 2** Correcting for internalizing problems at baseline, a three-way interaction effect of parental history severity, squared basal cortisol and chronic stress significantly predicted self-reported but not parent-reported internalizing problems.

|  | Parent-reported INTadj | | |  | Self-reported INTadj | | |
| --- | --- | --- | --- | --- | --- | --- | --- |
| Parameter | Estimate*^a^* | SE*^a^* | *p* |  | Estimate*^a^* | SE*^a^* | *p* |
| Intercept*^b^* | -68.50 | 176.20 | .70 |  | 473.84 | 194.71 | **.015** |
| Age | -7.25 | 8.00 | .37 |  | -20.83 | 8.89 | **.020** |
| Sex*^c^* | -76.57 | 32.80 | **.020** |  | -416.60 | 36.13 | **<.001** |
| Sampling month | 41.77 | 53.69 | .44 |  | 79.13 | 58.82 | .18 |
| Methylphenidate | 62.02 | 63.37 | .33 |  | -37.10 | 68.89 | .59 |
| Other psychotropics | 362.82 | 120.34 | **.003** |  | 234.01 | 129.04 | .07 |
| T1 INTadj | 495.45 | 16.84 | **<.001** |  | 342.11 | 17.74 | **<.001** |
| Stress | -17.79 | 84.71 | .83 |  | -126.69 | 93.92 | .18 |
| Cort | -4.71 | 21.88 | .83 |  | -11.72 | 24.08 | .63 |
| Cort² | 0.39 | 0.84 | .64 |  | 0.30 | 0.92 | .74 |
| PH | 42.10 | 153.68 | .78 |  | -81.64 | 167.48 | .63 |
| Cort*Stress | 31.48 | 14.61 | **.031** |  | 32.83 | 16.16 | **.042** |
| Cort²*Stress | -1.12 | 0.57 | **.050** |  | -0.96 | 0.63 | .13 |
| PH*Cort | 2.03 | 24.69 | .93 |  | 14.76 | 26.90 | .58 |
| PH*Cort² | -0.23 | 0.91 | .80 |  | -0.56 | 0.98 | .57 |
| PH*Stress | 133.15 | 84.50 | .12 |  | 226.55 | 93.32 | **.015** |
| PH*Cort*Stress | -21.64 | 14.19 | .13 |  | -36.32 | 15.65 | **.020** |
| PH*Cort²*Stress | 0.78 | 0.54 | .15 |  | 1.28 | 0.59 | **.031** |

*Note.* Cort = Awakening cortisol level; Cort² = Squared awakening cortisol level; PH = Parental history severity; INTadj = Internalizing problems adjusted for externalizing problems.

*^a^*Values multiplied by 1000 for increased readability.

*^b^*Participants varied significantly (*p<*.01) in intercept for parent-reported INTadj, var(*u*0*j*)=229.12*^a^*, chi-square(1)=187.60, and self-reported INTadj, var(*u*0*j*)=257.98*^a^*, chi-square(1)=168.03.

*^c^*Sex was coded as 0­ = *female*, 1 = *male*.

**Fig.1** Parent-reported (upper panel, *p* = .021) and self-reported (lower panel, *p* = .11) externalizing problem levels corrected for initial problem levels at T1, plotted for different levels of chronic stress and basal cortisol, and separately depicted for very severe PH (a) and no PH (b)


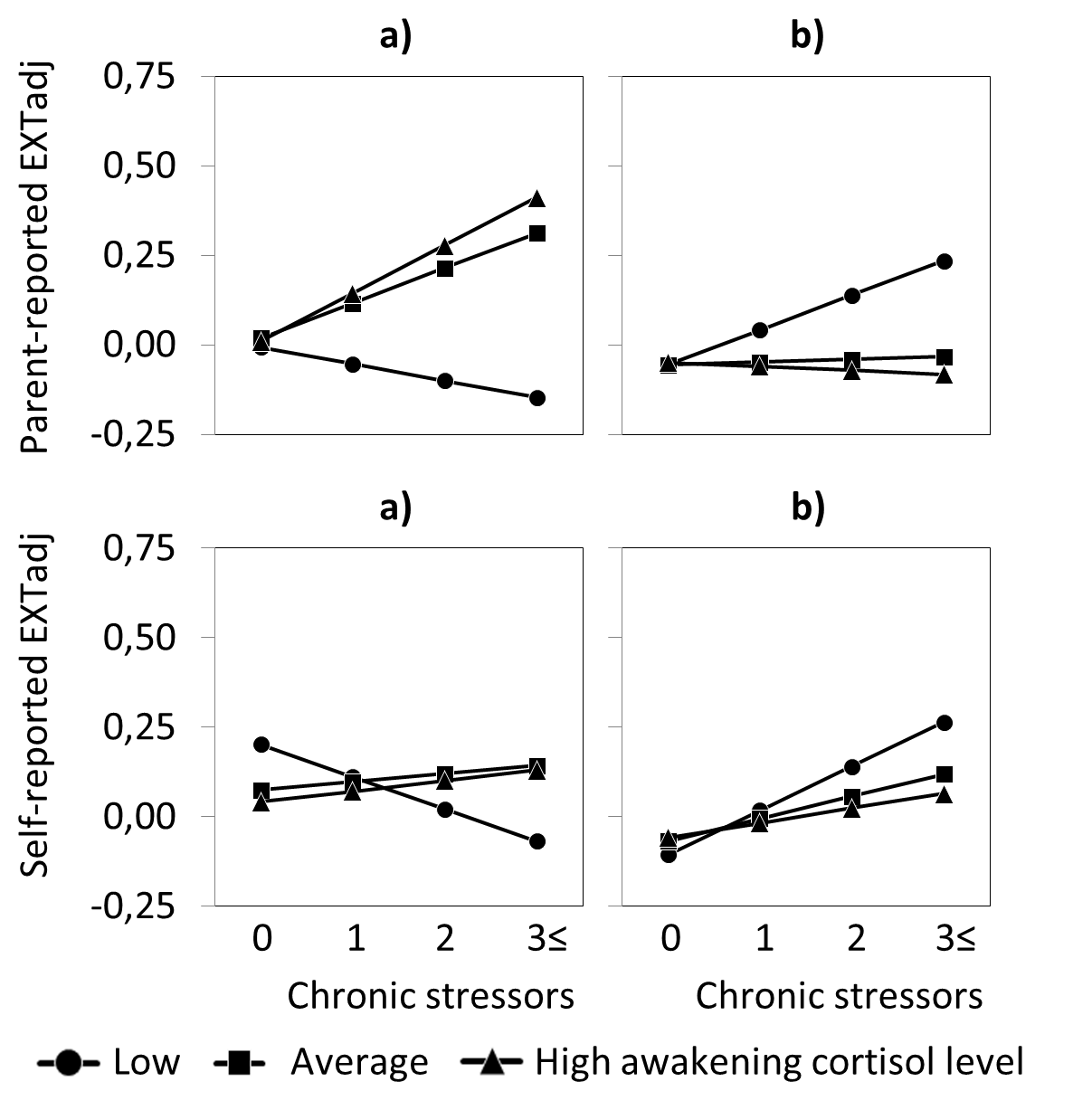


*Note*. PH = Parental history severity; EXTadj = Externalizing problems adjusted for internalizing problems. Levels of chronic stress refer to the number of long-term difficulties at T2. Low, average and high cortisol (-1SD, M, and +1SD) correspond to 6.15, 10.87, and 15.60 nmol/l, respectively.

**Fig.2** Parent-reported (left panel, *p* = .15) and self-reported (right panel, *p* = .031) internalizing problem levels corrected for initial problem levels at T1, plotted for different levels of chronic stress and basal cortisol, and separately depicted for very severe PH (a) and no PH (b)


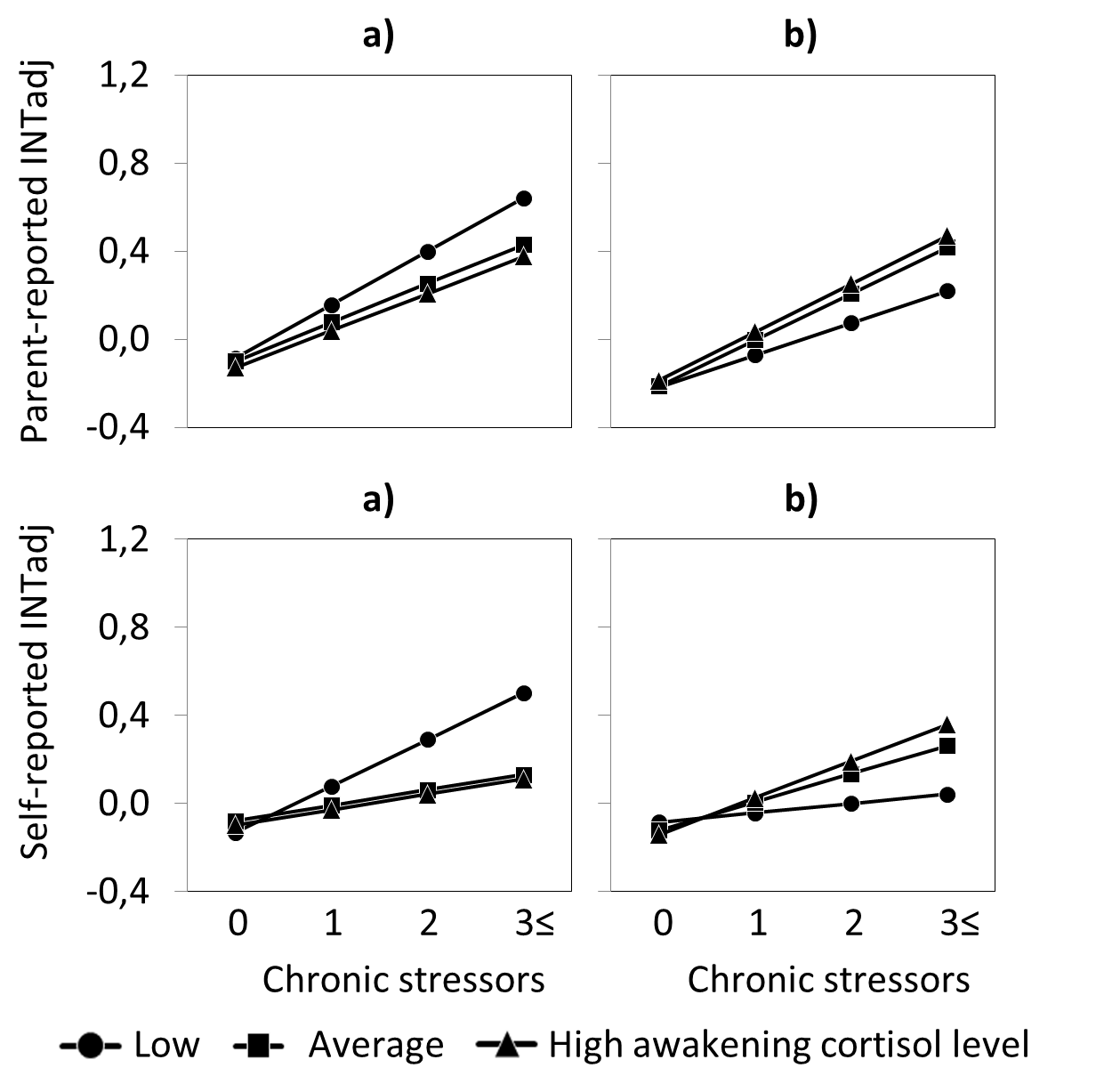


*Note*. PH = Parental history severity; INTadj = Internalizing problems adjusted for externalizing problems. Levels of chronic stress refer to the number of long-term difficulties at T2. Low, average and high cortisol (-1SD, M, and +1SD) correspond to 6.15, 10.87, and 15.60 nmol/l, respectively.
