# Supplementary material for: Chronic Stress and Adolescents’ Mental Health: Modifying Effects of Basal Cortisol and Parental Psychiatric History. The TRAILS Study
Source: J Abnorm Child Psychol. 2015 Jan 25;43(6):1119–30. doi: 10.1007/s10802-014-9970-x (PMC4494132; doi:10.1007/s10802-014-9970-x)
Supplement: Supplementary file 2 — (DOCX 191 kb) [file 10802_2014_9970_MOESM2_ESM.docx]

**Online Resource 2**

Tables and figures of externalizing and internalizing problems unadjusted for their co-occurrence.

This material is supplementary to:

Chronic stress and adolescents’ mental health: Modifying effects of basal cortisol and parental psychiatric history. The TRAILS study. *Journal of Abnormal Child Psychology.*

Anna Roos E. Zandstra, Catharina A. Hartman, Esther Nederhof, Edwin R. van den Heuvel, Andrea Dietrich, Pieter J. Hoekstra, Johan Ormel

Corresponding Author: Anna Roos Zandstra, Faculty of Mathematics and Natural Sciences, University of Groningen, The Netherlands. E-mail: A.R.E.Zandstra@rug.nl

**Table 1** Without adjusting externalizing problems for co-occurring internalizing problems, a three-way interaction effect of parental history severity, squared basal cortisol and chronic stress was marginally significant in predicting parent-reported externalizing problems and not significant in predicting self-reported externalizing problems.

|  | Parent-reported EXT | | |  | Self-reported EXT | | |
| --- | --- | --- | --- | --- | --- | --- | --- |
| Parameter | Estimate*^a^* | SE*^a^* | *p* |  | Estimate*^a^* | SE*^a^* | *p* |
| Intercept*^b^* | -337.58 | 181.39 | .06 |  | -217.94 | 210.41 | .30 |
| Age | 2.10 | 7.01 | .76 |  | -14.24 | 9.02 | .11 |
| Sex*^c^* | 92.85 | 39.00 | **.017** |  | 89.81 | 41.26 | **.030** |
| Sampling month | -130.44 | 64.33 | **.043** |  | -34.36 | 67.94 | .61 |
| Methylphenidate | 583.06 | 76.00 | **<.001** |  | 309.65 | 80.42 | **<.001** |
| Other psychotropics | -54.30 | 143.66 | **<.001** |  | -54.30 | 150.53 | .72 |
| Stress | 285.67 | 87.72 | **<.001** |  | 285.67 | 103.03 | **.006** |
| CORT | -26.34 | 24.67 | .29 |  | 30.79 | 27.18 | .26 |
| CORT² | 1.34 | 0.94 | .16 |  | -1.04 | 1.04 | .31 |
| PH | 228.57 | 171.20 | .18 |  | 384.36 | 188.83 | **.042** |
| CORT*Stress | -25.40 | 15.10 | .07 |  | -25.40 | 17.71 | .15 |
| CORT²*Stress | 0.75 | 0.59 | .20 |  | 0.84 | 0.69 | .23 |
| PH*CORT | 0.30 | 27.55 | .99 |  | -46.09 | 30.36 | .13 |
| PH*CORT² | -0.41 | 1.01 | .69 |  | 1.47 | 1.11 | .18 |
| PH*Stress | -222.73 | 87.86 | **.011** |  | -205.41 | 102.52 | **.045** |
| PH*CORT*Stress | 32.52 | 14.69 | **.027** |  | 28.51 | 17.16 | .10 |
| PH*CORT²*Stress | -1.00 | 0.56 | .07 |  | -0.98 | 0.65 | .13 |

*Note.* CORT = Awakening cortisol level; PH = Parental history severity; EXT = Externalizing problems unadjusted for co-occurring internalizing problems.

*^a^*Values multiplied by 1000 for increased readability.

*^b^*Participants varied significantly (*p<*.01) in intercept for parent-reported EXT, var(*u*0*j*)=509.64*^a^*, chi-square(1)=677.15, and self-reported EXT, var(*u*0*j*)=458.87*^a^*, chi-square(1)=342.89.

*^c^*Sex was coded as 0 = *female*, 1 = *male*.

**Table 2** Without adjusting internalizing problems for co-occurring externalizing problems, a three-way interaction effect of parental history severity, squared basal cortisol and chronic stress was not significant in predicting parent-reported internalizing problems but significantly predicted self-reported internalizing problems.

|  | Parent-reported INT | | |  | Self-reported INT | | |
| --- | --- | --- | --- | --- | --- | --- | --- |
| Parameter | Estimate*^a^* | SE*^a^* | *p* |  | Estimate*^a^* | SE*^a^* | *p* |
| Intercept*^b^* | -159.96 | 178.82 | .37 |  | 494.54 | 200.96 | **.014** |
| Age | -13.28 | 7.45 | .07 |  | -25.74 | 8.65 | **.003** |
| Sex*^c^* | -75.17 | 36.03 | **.037** |  | -472.04 | 39.26 | **<.001** |
| Sampling month | -68.22 | 59.41 | .25 |  | 25.43 | 64.64 | .69 |
| Methylphenidate | 423.49 | 70.31 | **<.001** |  | 101.20 | 76.52 | .19 |
| Other psychotropics | 1021.12 | 133.05 | **<.001** |  | 170.52 | 143.24 | .23 |
| Stress | 198.40 | 87.05 | **.023** |  | -73.06 | 98.37 | .46 |
| CORT | -19.93 | 23.43 | .40 |  | -10.50 | 25.90 | .69 |
| CORT² | 1.03 | 0.90 | .25 |  | 0.17 | 0.99 | .86 |
| PH | 239.98 | 163.70 | .14 |  | 13.02 | 179.99 | .94 |
| CORT*Stress | 21.32 | 14.99 | .16 |  | 34.14 | 16.91 | **.044** |
| CORT²*Stress | -0.87 | 0.59 | .14 |  | -1.02 | 0.66 | .12 |
| PH*CORT | -4.41 | 26.32 | .87 |  | 8.27 | 28.93 | .78 |
| PH*CORT² | -0.27 | 0.97 | .78 |  | -0.35 | 1.06 | .74 |
| PH*Stress | 81.88 | 87.05 | .35 |  | 191.31 | 97.88 | .05 |
| PH*CORT*Stress | -13.93 | 14.59 | .34 |  | -34.35 | 16.38 | **.036** |
| PH*CORT²*Stress | 0.51 | 0.55 | .36 |  | 1.26 | 0.62 | **.043** |

*Note.* CORT = Awakening cortisol level; PH = Parental history severity; INT = Internalizing problems unadjusted for co-occurring externalizing problems.

*^a^*Values multiplied by 1000 for increased readability.

*^b^*Participants varied significantly (*p <* .01) in intercept for parent-reported INT, var(*u*0*j*)=379.31*^a^*, chi-square(1)=412.88, and self-reported INT, var(*u*0*j*)=410.93*^a^*, chi-square(1)=332.27.

*^c^*Sex was coded as 0 = *female*, 1 = *male*.

**Fig.1** Parent-reported (upper panel, *p* = .07) and self-reported (lower panel, *p* = .13) externalizing problem levels unadjusted for co-occurring internalizing problems, plotted for different levels of chronic stress and basal cortisol, and separately depicted for very severe PH (a) and no PH (b)

*
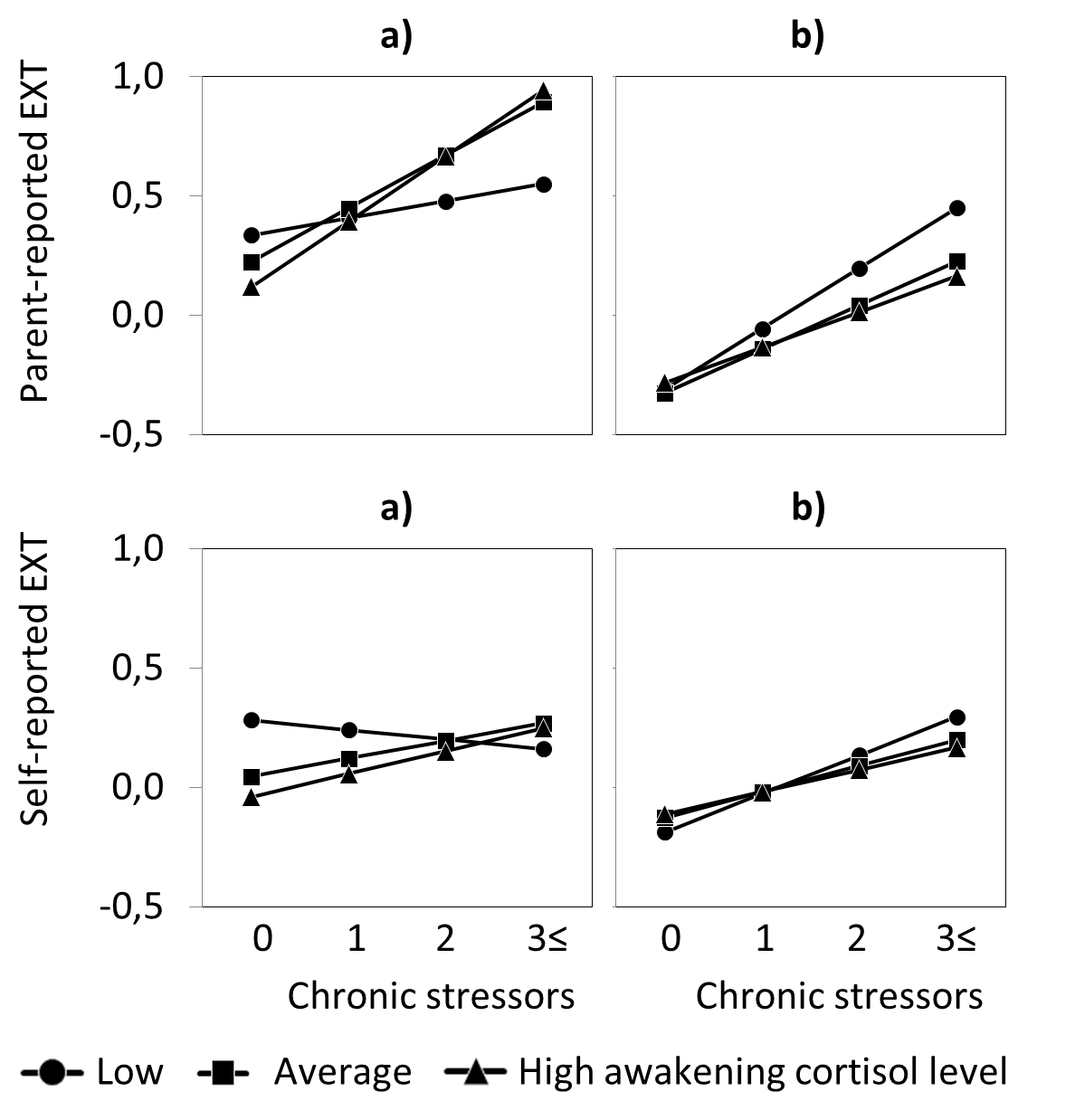
*

*Note*. PH = Parental history severity; EXT = Externalizing problems unadjusted for co-occurring internalizing problems. Levels of chronic stress refer to the number of long-term difficulties at T2. Low, average and high cortisol (-1SD, M, and +1SD) correspond to 6.15, 10.87, and 15.60 nmol/l, respectively.

**Fig.2** Parent-reported (upper panel, *p* = .36) and self-reported (lower panel, *p* = .043) internalizing problem levels unadjusted for co-occurring externalizing problems, plotted for different levels of chronic stress and basal cortisol, and separately depicted for very severe PH (a) and no PH (b)


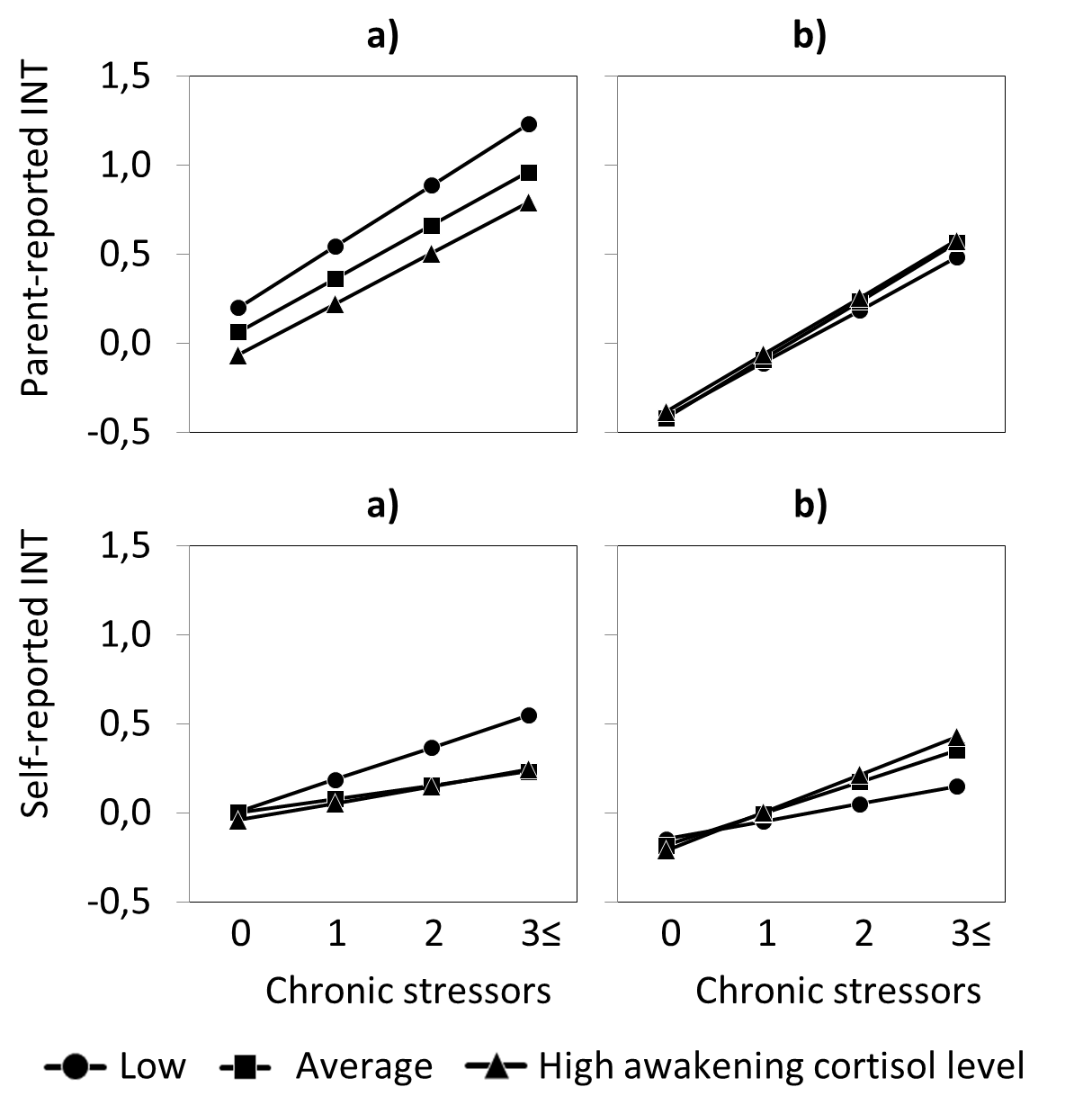


*Note*. PH = Parental history severity; INT = Internalizing problems unadjusted for co-occurring externalizing problems. Levels of chronic stress refer to the number of long-term difficulties at T2. Low, average and high cortisol (-1SD, M, and +1SD) correspond to 6.15, 10.87, and 15.60 nmol/l, respectively.
